# Supplementary material for: Markets as drivers of selection for highly virulent poultry pathogens
Source: Nat Commun. 2024 Jan 19;15:605. doi: 10.1038/s41467-024-44777-3 (PMC10799013; doi:10.1038/s41467-024-44777-3)
Supplement: Supplementary file 1 — Supplementary Information [file 41467_2024_44777_MOESM1_ESM.pdf]

Supporting Information for: "Markets as drivers for highly virulent poultry pathogens"
Nature Communications

Justin K. Sheen[1*], Fidisoa Rasambainarivo[1,2], Chadi Saad-Roy[3,4], Bryan T. Grenfell[1,5], and C. Jessica E. Metcalf[1,5]

[1]Department of Ecology and Evolutionary Biology, Princeton University, Princeton, NJ, USA
[2]Mahaliana Labs SARL, Antananarivo, Madagascar
[3]Miller Institute for Basic Research in Science, University of California, Berkeley, CA, USA
[4]Department of Integrative Biology, University of California, Berkeley, CA, USA
[5]School of Public and International Affairs, Princeton University, Princeton, NJ, USA
[*]Correspondence: Justin K. Sheen, jsheen@princeton.edu

December 2023

Appendix 1: Derivation of $\mathcal{R}_0$ of the market transmission model

We derive the $\mathcal{R}_0$ of a generalized version of our model that includes the potential for frequency-dependent transmission by including the parameter, p, where $p = 0$ makes transmission density-dependent and $p = 1$ makes transmission frequency-dependent:

$$\frac{dS}{dt} = b - \frac{\beta SI}{N^p} - \frac{\epsilon\beta SH}{N^p} - \mu S + m_f S_f - mS \tag{1a}$$

$$\frac{dE}{dt} = \frac{\beta SI}{N^p} + \frac{\epsilon\beta SH}{N^p} - \sigma E - \mu E - mE \tag{1b}$$

$$\frac{dI}{dt} = \sigma E - mI - \gamma I - \delta I - \mu I \tag{1c}$$

$$\frac{dR}{dt} = \gamma I - mR - \mu R \tag{1d}$$

$$\frac{dH}{dt} = \lambda - \kappa\psi H \tag{1e}$$

Following Heffernan et al. to create the next generation matrix, we first set up the F and V matrices.[1–3] We then substitute the DFE, $S[0]$, for S, as well as for N, since, $N[0] = S[0]$. For clarity, we do not further decompose $S[0]$ until the final step in calculating $\mathcal{R}_0$:

$$F = \begin{pmatrix} 0 & \frac{\beta}{(N^{[0]})^p} S^{[0]} & \frac{\epsilon\beta}{(N^{[0]})^p} S^{[0]} \\ 0 & 0 & 0 \\ 0 & 0 & 0 \end{pmatrix}; V = \begin{pmatrix} \sigma + \mu + m & 0 & 0 \\ -\sigma & m + \gamma + \delta + \mu & 0 \\ 0 & -\lambda & \kappa\psi \end{pmatrix}$$

$$F = \begin{pmatrix} 0 & \beta (S^{[0]})^{1-p} & \epsilon\beta (S^{[0]})^{1-p} \\ 0 & 0 & 0 \\ 0 & 0 & 0 \end{pmatrix}; V = \begin{pmatrix} \sigma + \mu + m & 0 & 0 \\ -\sigma & m + \gamma + \delta + \mu & 0 \\ 0 & -\lambda & \kappa\psi \end{pmatrix}$$

Next, we calculate V^{-1}:

$$V^{-1} = \frac{1}{|V|} \text{Ref} \begin{pmatrix} \kappa\psi(m + \gamma + \delta + \mu) & \sigma\kappa\psi & \lambda\sigma \\ 0 & \kappa\psi(\sigma + \mu + m) & \lambda(\sigma + \mu + m) \\ 0 & 0 & (\sigma + \mu + m)(m + \gamma + \delta + \mu) \end{pmatrix}$$

Let,

$$D = |V| = (\sigma + \mu + m)(\kappa\psi)(m + \gamma + \delta + \mu)$$

Then,

$$V^{-1} = \begin{pmatrix} \frac{\kappa\psi(m+\gamma+\delta+\mu)}{D} & 0 & 0 \\ \frac{\sigma\kappa\psi}{D} & \frac{\kappa\psi(\sigma+\mu+m)}{D} & 0 \\ \frac{\lambda\sigma}{D} & \frac{\lambda(\sigma+\mu+m)}{D} & \frac{(\sigma+\mu+m)(m+\gamma+\delta+\mu)}{D} \end{pmatrix}$$

Next, we find the next generation matrix, $G{=}FV^{-1}$:

$$G = FV^{-1} = \begin{pmatrix} 0 & \beta(S^{[0]})^{1-p} & \epsilon\beta(S^{[0]})^{1-p} \\ 0 & 0 & 0 \\ 0 & 0 & 0 \end{pmatrix} \begin{pmatrix} \frac{\kappa\psi(m+\gamma+\delta+\mu)}{D} & 0 & 0 \\ \frac{\sigma\kappa\psi}{D} & \frac{\kappa\psi(\sigma+\mu+m)}{D} & 0 \\ \frac{\lambda\sigma}{D} & \frac{\lambda(\sigma+\mu+m)}{D} & \frac{(\sigma+\mu+m)(m+\gamma+\delta+\mu)}{D} \end{pmatrix}$$

$$= \begin{pmatrix} \beta(S^{[0]})^{1-p}\frac{\sigma\kappa\psi}{D} + \epsilon\beta(S^{[0]})^{1-p}\frac{\lambda\sigma}{D} & \beta(S^{[0]})^{1-p}\frac{(\kappa\psi)(\sigma+\mu+m)}{D} + \epsilon\beta(S^{[0]})^{1-p}\frac{\lambda(\sigma+\mu+m)}{D} & \epsilon\beta(S^{[0]})^{1-p}\frac{(\sigma+\mu+m)(m+\gamma+\delta+\mu)}{D} \\ 0 & 0 & 0 \\ 0 & 0 & 0 \end{pmatrix}$$

Let,

$$G = \begin{pmatrix} e_1 & e_2 & e_3 \\ 0 & 0 & 0 \\ 0 & 0 & 0 \end{pmatrix}$$

Finally, we find the dominant eigenvalue of G by solving $|G - \lambda I| = 0$ (note that λ_1 and λ_2 are different from the shedding parameter, λ):

$$|G - \lambda I| = | \begin{pmatrix} e_1 - \lambda & e_2 & e_3 \\ 0 & \lambda & 0 \\ 0 & 0 & -\lambda \end{pmatrix} |$$
$$= (e_1 - \lambda)(\lambda^2) = 0$$
$$\lambda_1 = 0; \lambda_2 = e_1 = \beta(S^{[0]})^{1-p}\frac{\sigma\kappa\psi}{D} + \epsilon\beta(S^{[0]})^{1-p}\frac{\lambda\sigma}{D}$$

$\mathcal{R}_0$ is equal to the dominant eigenvalue, λ_2:

$$\mathcal{R}_0 = \frac{\beta(S^{[0]})^{1-p}\kappa\psi\sigma + \epsilon\beta(S^{[0]})^{1-p}\lambda\sigma}{(\sigma+\mu+m)(\kappa\psi)(m+\gamma+\delta+\mu)}$$
$$= \frac{(S^{[0]})^{1-p}(\beta\kappa\psi\sigma + \epsilon\beta\lambda\sigma)}{(\sigma+\mu+m)(\kappa\psi)(m+\gamma+\delta+\mu)}$$

The Disease-Free Equilibrium (DFE), $S^{[0]}$, is (where $N^{[0]} = S^{[0]}$):

$$\frac{dS}{dt} = b - \frac{\beta}{N^p}SI - \frac{\epsilon\beta}{N^p}SH - \mu S + m_f S_f - mS$$
$$0 = b - \mu S^{[0]} + m_f S_f - mS^{[0]}$$
$$S^{[0]}(\mu + m) = b + m_f S_f$$
$$S^{[0]} = \frac{b + m_f S_f}{\mu + m} = N^{[0]}$$

Thus, substituting for $S^{[0]}$:

$$\mathcal{R}_0 = \frac{(\frac{b+m_f S_f}{\mu+m})^{1-p}(\beta\kappa\psi\sigma + \epsilon\beta\lambda\sigma)}{(\sigma+\mu+m)(\kappa\psi)(m+\gamma+\delta+\mu)}$$

When the birth rate is 0, as in markets, $\mathcal{R}_0$ further simplifies to:

$$\mathcal{R}_0 = \frac{(\frac{m_f S_f}{\mu+m})^{1-p}(\beta\kappa\psi\sigma + \epsilon\beta\lambda\sigma)}{(\sigma+\mu+m)(\kappa\psi)(m+\gamma+\delta+\mu)}$$

We can rearrange the $\mathcal{R}_0$ equation:

$$\mathcal{R}_0 = \Big(\frac{m_f S_f}{\mu + m}\Big)^{1-p}\Big(\frac{\sigma}{\sigma + \mu + m}\Big)\Big(\frac{1}{m + \gamma + \delta + \mu}\Big)\beta\Big[1 + \frac{\epsilon\lambda}{\kappa\psi}\Big] \tag{2}$$

When transmission is frequency-dependent ($p = 1$), $\mathcal{R}_0$ further simplifies to:

$$\mathcal{R}_0 = \Big(\frac{\sigma}{\sigma + \mu + m}\Big)\Big(\frac{1}{m + \gamma + \delta + \mu}\Big)\beta\Big[1 + \frac{\epsilon\lambda}{\kappa\psi}\Big] \tag{3}$$

When transmission is frequency-dependent, $\mathcal{R}_0$ does not depend on m_f, S_f, μ, or b.

Appendix 2: Proof of validity of $\mathcal{R}_0$ maximization approach for adaptive dynamics when transmission is density-dependent

First, we find the endemic equilibrium of the resident. To do this, we set all model equations that describe the infection dynamics to 0. Then we put E^* in terms of I^* using the $\frac{dI}{dt} = 0$ equation (where * denotes the endemic equilibrium):

$$\sigma E^* = (m + \gamma + \delta + \mu)I^*$$
$$E^* = \frac{(m + \gamma + \delta + \mu)I^*}{\sigma}$$

We then put H^* in terms of I^* using the $\frac{dH}{dt} = 0$ equation:

$$\lambda I^* - \kappa\psi H^* = 0$$
$$H^* = \frac{\lambda I^*}{\kappa\psi}$$

Next, we use the $\frac{dE}{dt} = 0$ equation to find an expression for S^*:

$$\begin{aligned}
0 &= \beta S^* I^* + \epsilon\beta S^* H^* - (\sigma + \mu + m)E^* \\
&= \beta S^* I^* + \epsilon\beta S^* (\frac{\lambda}{\kappa\psi})I^* - (\sigma + \mu + m)(\frac{m + \gamma + \delta + \mu}{\sigma})I^* \\
I^* S^* (\beta + \epsilon\beta\frac{\lambda}{\kappa\psi}) &= (\sigma + \mu + m)(\frac{m + \gamma + \delta + \mu}{\sigma})I^* \\
S^* (\beta + \epsilon\beta\frac{\lambda}{\kappa\psi}) &= (\sigma + \mu + m)(\frac{m + \gamma + \delta + \mu}{\sigma}) \\
S^* &= (\sigma + \mu + m)(\frac{m + \gamma + \delta + \mu}{\sigma})\frac{1}{(\beta + \epsilon\beta\frac{\lambda}{\kappa\psi})}
\end{aligned}$$

Next, we find $\frac{1}{S^*}$:

$$\begin{aligned}
S^* &= (\sigma + \mu + m)(\frac{m + \gamma + \delta + \mu}{\sigma})\frac{1}{(}\beta + \epsilon\beta\frac{\lambda}{\kappa\psi}) \\
\frac{1}{S^*} &= \frac{\sigma(\beta + \epsilon\beta\frac{\lambda}{\kappa psi})}{(\sigma + \mu + m)(m + \gamma + \delta + \mu)} \\
&= \frac{\sigma}{\sigma + \mu + m}[(\beta + \epsilon\beta\frac{\lambda}{\kappa\psi})\frac{1}{m + \gamma + \delta + \mu}] \\
&= (\frac{\sigma}{\sigma + \mu + m})(\frac{1}{m + \gamma + \delta + \mu})\beta[1 + \frac{\epsilon\lambda}{\kappa\psi}] \\
&= \frac{\mathcal{R}_0}{S^{[0]}}
\end{aligned}$$

Thus,

$$S^* = \frac{S^{[0]}}{\mathcal{R}_0} \tag{4}$$

$$S_r^* = \frac{S^{[0]}}{\mathcal{R}_0(\alpha_r)} \tag{5}$$

The differential equations of the mutant strategy that tries to invade the resident population when initially rare are:

$$\begin{aligned}
\frac{dE}{dt} &= \beta S_r^* + \epsilon\beta S_r^* H - \sigma E - \mu E - mE \\
\frac{dI}{dt} &= \sigma E - mI - \gamma I - \delta I - \mu I \\
\frac{dH}{dt} &= \lambda I - \kappa\psi H
\end{aligned}$$

In order to find an expression for $\mathcal{R}_i(\alpha_r, \alpha_m)$, which is written as $s_r(m)$ in "The Hitchhiker's Guide to Adaptive Dynamics," or the invasion fitness of the mutant when it tries to invade the resident, we use a next-generation approach.[4] First, we find that the F and V matrices for the next-generation matrix G are:

$$F = \begin{pmatrix} 0 & \beta S_r^* & \epsilon\beta S_r^* \\ 0 & 0 & 0 \\ 0 & 0 & 0 \end{pmatrix}; V = \begin{pmatrix} \sigma + \mu + m & 0 & 0 \\ -\sigma & m + \gamma + \delta + \mu & 0 \\ 0 & -\lambda & \kappa\psi \end{pmatrix}$$

Immediately, we recognize that the only difference between the F and V matrices here and the F and V matrices when deriving $\mathcal{R}_0$ above is the replacement of $S^{[0]}$ with S_r^*. Thus, because the rest of the derivation is the same as the above, to arrive at an expression for $\mathcal{R}_i(\alpha_r, \alpha_m)$ we can simply replace $S^{[0]}$ with S_r^*. Thus, because the rest of the derivation is the same as the above, to arrive at an expression for $\mathcal{R}_i(\alpha_r, \alpha_m)$ we can simply replace $S^{[0]}$ with S_r^* in the expression of $\mathcal{R}_0$:

$$\begin{aligned}
\mathcal{R}_i(\alpha_r, \alpha_m) &= S_r^*(\frac{\sigma}{\sigma + \mu + m})(\frac{1}{m + \gamma + \delta + \mu})\beta[1 + \frac{\epsilon\lambda}{\kappa\psi}] \\
&= S_r^* \frac{\mathcal{R}_0(\alpha_m)}{S^{[0]}}
\end{aligned}$$

Next, we plug S_r^* into our expression for $\mathcal{R}_i(\alpha_r, \alpha_m)$:

$$\begin{aligned}
\mathcal{R}_i(\alpha_r, \alpha_m) &= S_r^* \frac{\mathcal{R}_0(\alpha_m)}{S^{[0]}} \\
&= \frac{S^{[0]}}{\mathcal{R}_0(\alpha_r)} \frac{\mathcal{R}_0(\alpha_m)}{S^{[0]}} \\
&= \frac{\mathcal{R}_0(\alpha_m)}{\mathcal{R}_0(\alpha_r)}
\end{aligned} \tag{6}$$

Next, we look for singular strategies (critical points of $\mathcal{R}_i(\alpha_r, \alpha_m)$). We do this by taking the derivative of $\mathcal{R}_i(\alpha_r, \alpha_m)$ with respect to the mutant strategy, then evaluating it at $\alpha_m = \alpha_r$ and setting it to 0:[4]

$$\begin{aligned}
\frac{\partial}{\partial \alpha_m}\mathcal{R}_i(\alpha_r, \alpha_m) &= \frac{\mathcal{R}_0'(\alpha_m)}{\mathcal{R}_0(\alpha_r)} \\
0 &= \frac{\mathcal{R}_0'(\alpha_m)}{\mathcal{R}_0(\alpha_r)} \\
0 &= \frac{\mathcal{R}_0'(\alpha_m = \alpha_r)}{\mathcal{R}_0(\alpha_r)} \\
0 &= \frac{\mathcal{R}_0'(\alpha_r)}{\mathcal{R}_0(\alpha_r)}
\end{aligned}$$

Thus, singular strategies are those virulence strategies, α^*, such that $\mathcal{R}_0' = 0$ since the denominator is positive.

Next, we check the condition for convergence stability of the singular strategy, which is:

$$\begin{aligned}
\frac{\partial}{\partial \alpha_r}\mathcal{R}_i'(\alpha_r, \alpha_m = \alpha_r)\Big|_{\alpha_r = \alpha^*} &< 0 \qquad (7) \\
\frac{\partial}{\partial \alpha_r}\frac{\mathcal{R}_0'(\alpha_r)}{\mathcal{R}_0(\alpha_r)}\Big|_{\alpha_r = \alpha^*} &< 0 \\
\frac{\mathcal{R}_0''(\alpha_r)}{\mathcal{R}_0(\alpha_r)} - \frac{(\mathcal{R}_0'(\alpha_r))^2}{(\mathcal{R}_0(\alpha_r))^2}\Big|_{\alpha_r = \alpha^*} &< 0 \\
\frac{\mathcal{R}_0''(\alpha^*)}{\mathcal{R}_0(\alpha^*)} - \frac{(\mathcal{R}_0'(\alpha^*))^2}{(\mathcal{R}_0(\alpha^*))^2} &< 0 \\
\frac{\mathcal{R}_0''(\alpha^*)}{\mathcal{R}_0(\alpha^*)} &< 0
\end{aligned}$$

In the fourth line, the second term on the L.H.S. equals 0 since $\mathcal{R}_0'(\alpha^*) = 0$ since α^* is a singular strategy (critical point). Thus, for convergence stability, the condition is that $\mathcal{R}''(\alpha^*) < 0$ since the denominator is positive. This is exactly the ESS criterion of the singular strategy, since:[4]

$$\begin{aligned}
\frac{\partial^2 \mathcal{R}_i(\alpha_r, \alpha_m)}{\partial \alpha_m^2}\Big|_{\alpha_r = \alpha_m = \alpha^*} &< 0 \qquad (8) \\
\frac{\mathcal{R}_0''(\alpha^*)}{\mathcal{R}_0(\alpha^*)} &< 0
\end{aligned}$$

And thus, the ESS criterion is that $\mathcal{R}_0''(\alpha^*) < 0$ since the denominator is positive.

We have shown that the ESS criterion and condition for convergence stability of a singular strategy is the same. Furthermore, α^* such that $\mathcal{R}_0''(\alpha^*) < 0$ are local maxima of $\mathcal{R}_0$ and thus if a virulence strategy is evolutionary stable and convergent stable then it is a local maxima of $\mathcal{R}_0$. In the other direction, if a virulence strategy is a local maxima of $\mathcal{R}_0$ this

implies that it is evolutionarily stable and convergent stable. Thus, there is an if-and-only-if condition of local maxima of $\mathcal{R}_0$ and evolutionary stability and convergence stability. Thus, by finding the virulence strategy that maximizes $\mathcal{R}_0$, this is equivalent to finding the singular strategy that is both evolutionarily stable and convergent stable.

Appendix 3: Additional sensitivity test results, justifications, and figures

When there is no relationship between virulence and shedding rate, λ, the global ESS will not change depending on changes in λ

The expression for $\mathcal{R}_0$ (Eq. 2) is:

$$\mathcal{R}_0 = \Big(\frac{m_f S_f}{\mu + m}\Big)^{1-p}\Big(\frac{\sigma}{\sigma + \mu + m}\Big)\Big(\frac{1}{m + \gamma + \delta + \mu}\Big)\beta\Big[1 + \frac{\epsilon\lambda}{\kappa\psi}\Big]$$

λ solely appears in the last factor. When λ is not a function of virulence, α, λ only affects the scaling factor of $\mathcal{R}_0$ and thus does not affect the shape of the fitness landscape of $\mathcal{R}_0$ vs. α and thus the position of the global ESS.

Full results $\phi = 0.1, 1, 10,$ or 100

% of tradeoff curves with decrease in global ESS;
max decrease in global ESS from baseline market conditions

ϕ	N tradeoff curves	decrease m and increase κ	decrease solely m	decrease solely κ
0.1	19	79%; −499	79%; −474	47%; −76
1	18	78%; −499	72%; −474	72%; −81
10	12	75%; −722	67%; −266	75%; −456
100	8	63%; −759	63%; −485	63%; −587

Table S1: **The percent of tradeoff curves with a decrease in the global ESS; the maximum decrease in global ESS.** The majority of tradeoff curves show a decrease in the global ESS when decreasing both m and κ, decreasing solely m, and decreasing solely κ (with the exception of when $\phi = 0.1$ when decreasing solely κ). There are no increases in global ESS.

Sensitivity test: infectious poultry emigrate at a 50% slower rate compared to all other poultry

% of tradeoff curves with decrease in global ESS;
max decrease in global ESS from baseline market conditions

ϕ	N tradeoff curves	decrease m and increase κ	decrease solely m	decrease solely κ
0.1	17	76%; −258	76%; −233	35%; −47
1	15	73%; −496	67%; −233	67%; −263
10	12	75%; −724	67%; −131	75%; −593
100	9	67%; −724	67%; −237	63%; −593

Table S2: **The percent of tradeoff curves with a decrease in the global ESS; the maximum decrease in global ESS when infectious poultry emigrate 50% slower from markets compared to all other poultry.** The majority of tradeoff curves show a decrease in the global ESS when decreasing both m and κ, decreasing solely m, and decreasing solely κ (with the exception of when $\phi = 0.1$ when decreasing solely κ). With differential migration, the maximum decrease in the global ESS due to decreases in m becomes smaller, and the maximum decrease in global ESS due to increases in κ generally becomes larger (with the exception of when $\phi = 0.1$). There are no increases in global ESS.

Justification for range of c_1 and c_2

Param,	Description	Value	Reference
N_f^0	Number of poultry in farms that use markets	1 million	Roughly twice as much as a coastal region in Tanzania, Chuma et al.[5]
N_0	initial number of poultry located in market	100	assumed
ϕ	prevalence of infected but not infectious (E) and infectious (I) poultry in farms	12%	Mngumi et al.[6]
ρ	seroprevalence of the pathogen in farms	40.2%	Mngumi et al.[6]

Table S3: **Parameters for initial compartment numbers.** The prevalence and seroprevalence estimates come from studies of Newcastle disease virus. In order to get the number of susceptible, exposed, infected, and recovered poultry in farms, we first multiply N_0^f by the prevalence among unvaccinated apparently healthy birds, ϕ, which we assume are still in the incubation period, or on the cusp of the symptomatic infectious period. For simplification we divide this number evenly into the number of infected but not infectious (E), and infectious (I) poultry. We assume that the seroprevalence was sampled from S, E, and R, poultry, since unvaccinated apparently healthy birds were sampled. Therefore $\rho = R/(S + E + R)$ and $R = \rho(S + E + R)$. To get the denominator, we first observe $S + R = (1 - \phi)$ times N_0^f and add it to the number of E poultry, which was already solved for above, to get $S + E + R$. We multiply ρ by this quantity to get the number of R poultry. To get the number of S poultry, we then simply calculate $S = N_0^f$ - E - I - R in the above.

We first get the initial number of S poultry using the parameters in Table S3. To get the ranges of c_1 and c_2: we first note that c_2 defines how flat the relationship is between transmission and mortality. We choose the minimum value of 0.1 as the flattest value. We choose the maximum value of 1 since this leads to a completely linear relationship between transmission and mortality, and we are interested in concave-down relationships. Next, we choose c_1, which defines how much transmission is conferred per virulence step. We find the approximate minimum c_1 when $c_2 = 0.1$ or 1 such that at least one poultry will be infected by an infectious poultry, and choose the maximum c_1 when $c_2 = 0.1$ or 1, such that no greater than two hundred poultry will be infected by an infectious poultry. These bounds of no less than one poultry infected and no greater than two hundred poultry infected are defined when infection occurs in a susceptible pool of poultry with population size equal to the disease free equilibrium when transmission is density dependent, which in markets $= \frac{m_f S_f}{\mu + m} = 2267.22$. The minimum we find is 1/2250000 and the maximum we find is 1/23.

Justification of comparison of selection pressures in markets vs. farms

Characteristic	Relative comparison in farms vs. markets	Reference
Turnover rate (m)	This will be faster for markets since the turnover rate is very fast. In farms, poultry live on average one year.	data collected in Madagascar
Birth rate (b)	Since there are negligible births of poultry in markets and nearly all new poultry in farms are self-hatched, this should be higher in farms.	McCarron et al.[7]
Inflow of susceptible poultry (m_f and S_f)	Since nearly all new poultry in farms are self-hatched, this should be lower in farms.	McCarron et al.[7]
Contact rate (affects β)	Markets may have higher contact rates than farms since poultry are housed in close quarters with large population sizes.	McCarron et al.[7]
Cleanliness of the environment (κ)	Markets may be hypothesized to have less clean conditions relative to farms since there is less incentive to keep poultry alive since there is fast turnover. There is also observational evidence of the unhygienic conditions of the cages these poultry are kept in during transport to markets.	Kariithi et al.,[8] Molina et al.[9]
Local vs. global transmission	Due to the lower mean flock size in farms compared to markets there may be more local structure in farms, compared to markets, which have smaller populations with repeated contacts and stochastic extinctions.	Okeno et al.,[10] McCarron et al.[7]

Table S4: **Relative comparisons of key characteristics in farms vs. markets that may shape selection pressures for the evolution of virulence.** We note the relative differences in key characteristics between farms vs. markets that may lead to differing selection pressures for the evolution of virulence for viral poultry pathogens. When possible, we list the parameter in our model that corresponds with each characteristic. According to our model, of the first five characteristics, m and κ are hypothesized to have an effect on the selection of the global ESS virulence (see "Justification of comparison of selection pressures in markets vs. farms"). The last characteristic, local vs. global transmission, is not captured by our model, but may further create differences between the global ESS virulence in farms vs. markets (see Discussion).

Comparing selection pressures in markets vs. farms, requires exploring how the optimal virulence strategy (the global ESS) changes in response to changes in the parameters of our transmission model (see "Transmission Assumptions in Markets" and Figure 2) to market conditions vs. farm conditions. Under farm conditions, the turnover rate of poultry into the system, m, would be lower, the rate of migration of poultry into the system and number

of poultry that migrate into the system, m_f and S_f, respectively, would be lower, κ would increase due to cleaner conditions, the birth rate, b, would be non-zero, and the transmission rate β may be lower (Table S4). However, of these parameters, only m and κ will potentially change the shape of the fitness landscape dependent on virulence, and thus change the position of the global ESS: b is not included in the $\mathcal{R}_0$ expression, and changes in m_f, S_f, and β scale $\mathcal{R}_0$ and would not change the position of the global ESS. Thus, the relative differences in turnover rate and κ will allow us to make a qualitative comparison between the selection pressures in farms vs. markets. Table S4 compares these characteristics in the two settings.

Appendix 4: Sensitivity analysis when there is differential turnover of infectious poultry

We perform a sensitivity analysis of our results when there is differential turnover of infectious poultry. The model equations are:

$$\frac{dS}{dt} = b - \frac{\beta SI}{N^p} - \frac{\epsilon\beta SH}{N^p} - \mu S + m_f S_f - mS \tag{9a}$$
$$\frac{dE}{dt} = \frac{\beta SI}{N^p} + \frac{\epsilon\beta SH}{N^p} - \sigma E - \mu E - mE \tag{9b}$$
$$\frac{dI}{dt} = \sigma E - m_I I - \gamma I - \delta I - \mu I \tag{9c}$$
$$\frac{dR}{dt} = \gamma I - mR - \mu R \tag{9d}$$
$$\frac{dH}{dt} = \lambda I - \kappa\psi H \tag{9e}$$

Where $m_I = 0.5 \times m$, i.e., infectious poultry, who are more symptomatic, have a turnover rate 50% slower than all other poultry out of markets. This models accounts for the case when transmission is frequency-dependent ($p = 1$) or density-dependent ($p = 0$). We assume in this model that infected but not infectious poultry are not symptomatic, and thus will migrate at the same rate as susceptible and recovered poultry.

Derivation of $\mathcal{R}_0$ of the market transmission model when there is differential turnover of infectious poultry

Following Heffernan et al. to create the next generation matrix, we first set up the F and V matrices.[1–3] We then substitute the DFE, $S[0]$, for S, as well as for N, since, $N[0] = S[0]$. For clarity, we do not further decompose $S[0]$ until the final step in calculating $\mathcal{R}_0$:

$$F = \begin{pmatrix} 0 & \frac{\beta}{(N^{[0]})^p} S^{[0]} & \frac{\epsilon\beta}{(N^{[0]})^p} S^{[0]} \\ 0 & 0 & 0 \\ 0 & 0 & 0 \end{pmatrix}; V = \begin{pmatrix} \sigma + \mu + m & 0 & 0 \\ -\sigma & m_I + \gamma + \delta + \mu & 0 \\ 0 & -\lambda & \kappa\psi \end{pmatrix}$$
$$F = \begin{pmatrix} 0 & \beta(S^{[0]})^{1-p} & \epsilon\beta(S^{[0]})^{1-p} \\ 0 & 0 & 0 \\ 0 & 0 & 0 \end{pmatrix}; V = \begin{pmatrix} \sigma + \mu + m & 0 & 0 \\ -\sigma & m_I + \gamma + \delta + \mu & 0 \\ 0 & -\lambda & \kappa\psi \end{pmatrix}$$

Next, we calculate V^{-1}:

$$V^{-1} = \frac{1}{|V|}\text{Ref}\begin{pmatrix} \kappa\psi(m_I + \gamma + \delta + \mu) & \sigma\kappa\psi & \lambda\sigma \\ 0 & \kappa\psi(\sigma + \mu + m) & \lambda(\sigma + \mu + m) \\ 0 & 0 & (\sigma + \mu + m)(m_I + \gamma + \delta + \mu) \end{pmatrix}$$

Let,

$$D = |V| = (\sigma + \mu + m)(\kappa\psi)(m_I + \gamma + \delta + \mu)$$

Then,

$$V^{-1} = \begin{pmatrix} \frac{\kappa\psi(m_I+\gamma+\delta+\mu)}{D} & 0 & 0 \\ \frac{\sigma\kappa\psi}{D} & \frac{\kappa\psi(\sigma+\mu+m)}{D} & 0 \\ \frac{\lambda\sigma}{D} & \frac{\lambda(\sigma+\mu+m)}{D} & \frac{(\sigma+\mu+m)(m_I+\gamma+\delta+\mu)}{D} \end{pmatrix}$$

Next, we find the next generation matrix, $G{=}FV^{-1}$:

$$\begin{aligned} G = FV^{-1} &= \begin{pmatrix} 0 & \beta(S^{[0]})^{1-p} & \epsilon\beta(S^{[0]})^{1-p} \\ 0 & 0 & 0 \\ 0 & 0 & 0 \end{pmatrix} \begin{pmatrix} \frac{\kappa\psi(m_I+\gamma+\delta+\mu)}{D} & 0 & 0 \\ \frac{\sigma\kappa\psi}{D} & \frac{\kappa\psi(\sigma+\mu+m)}{D} & 0 \\ \frac{\lambda\sigma}{D} & \frac{\lambda(\sigma+\mu+m)}{D} & \frac{(\sigma+\mu+m)(m_I+\gamma+\delta+\mu)}{D} \end{pmatrix} \\ &= \begin{pmatrix} \beta(S^{[0]})^{1-p}\frac{\sigma\kappa\psi}{D} + \epsilon\beta(S^{[0]})^{1-p}\frac{\lambda\sigma}{D} & \beta(S^{[0]})^{1-p}\frac{(\kappa\psi)(\sigma+\mu+m)}{D} + \epsilon\beta(S^{[0]})^{1-p}\frac{\lambda(\sigma+\mu+m)}{D} & \epsilon\beta(S^{[0]})^{1-p}\frac{(\sigma+\mu+m)(m_I+\gamma+\delta+\mu)}{D} \\ 0 & 0 & 0 \\ 0 & 0 & 0 \end{pmatrix} \end{aligned}$$

Let,

$$G = \begin{pmatrix} e_1 & e_2 & e_3 \\ 0 & 0 & 0 \\ 0 & 0 & 0 \end{pmatrix}$$

Finally, we find the dominant eigenvalue of G by solving $|G - \lambda I| = 0$ (note that λ_1 and λ_2 are different from the shedding parameter, λ):

$$\begin{aligned} |G - \lambda I| &= | \begin{pmatrix} e_1 - \lambda & e_2 & e_3 \\ 0 & \lambda & 0 \\ 0 & 0 & -\lambda \end{pmatrix} | \\ &= (e_1 - \lambda)(\lambda^2) = 0 \\ \lambda_1 &= 0; \lambda_2 = e_1 = \beta(S^{[0]})^{1-p}\frac{\sigma\kappa\psi}{D} + \epsilon\beta(S^{[0]})^{1-p}\frac{\lambda\sigma}{D} \end{aligned}$$

$\mathcal{R}_0$ is equal to the dominant eigenvalue, λ_2:

$$\begin{aligned} \mathcal{R}_0 &= \frac{\beta(S^{[0]})^{1-p}\kappa\psi\sigma + \epsilon\beta(S^{[0]})^{1-p}\lambda\sigma}{(\sigma+\mu+m)(\kappa\psi)(m_I+\gamma+\delta+\mu)} \\ &= \frac{(S^{[0]})^{1-p}(\beta\kappa\psi\sigma + \epsilon\beta\lambda\sigma)}{(\sigma+\mu+m)(\kappa\psi)(m_I+\gamma+\delta+\mu)} \end{aligned}$$

The Disease-Free Equilibrium (DFE), $S^{[0]}$, is (where $N^{[0]} = S^{[0]}$):

$$\begin{aligned} \frac{dS}{dt} &= b - \frac{\beta}{N^p}SI - \frac{\epsilon\beta}{N^p}SH - \mu S + m_f S_f - mS \\ 0 &= b - \mu S^{[0]} + m_f S_f - mS^{[0]} \\ S^{[0]}(\mu + m) &= b + m_f S_f \\ S^{[0]} &= \frac{b + m_f S_f}{\mu + m} = N^{[0]} \end{aligned}$$

Thus, substituting for $S^{[0]}$:

$$\mathcal{R}_0 = \frac{(\frac{b+m_f S_f}{\mu+m})^{1-p}(\beta\kappa\psi\sigma + \epsilon\beta\lambda\sigma)}{(\sigma+\mu+m)(\kappa\psi)(m_I+\gamma+\delta+\mu)}$$

When the birth rate is 0, as in markets, $\mathcal{R}_0$ further simplifies to:

$$\mathcal{R}_0 = \frac{(\frac{m_f S_f}{\mu+m})^{1-p}(\beta\kappa\psi\sigma + \epsilon\beta\lambda\sigma)}{(\sigma+\mu+m)(\kappa\psi)(m_I+\gamma+\delta+\mu)}$$

We can rearrange the $\mathcal{R}_0$ equation:

$$\mathcal{R}_0 = \Big(\frac{m_f S_f}{\mu+m}\Big)^{1-p}\Big(\frac{\sigma}{\sigma+\mu+m}\Big)\Big(\frac{1}{m_I+\gamma+\delta+\mu}\Big)\beta\Big[1+\frac{\epsilon\lambda}{\kappa\psi}\Big] \tag{10}$$

When transmission is frequency-dependent ($p = 1$), $\mathcal{R}_0$ further simplifies to:

$$\mathcal{R}_0 = \Big(\frac{\sigma}{\sigma+\mu+m}\Big)\Big(\frac{1}{m_I+\gamma+\delta+\mu}\Big)\beta\Big[1+\frac{\epsilon\lambda}{\kappa\psi}\Big] \tag{11}$$

When transmission is frequency-dependent, $\mathcal{R}_0$ does not depend on m_f, S_f, μ, or b.

Proof of validity of $\mathcal{R}_0$ maximization approach for adaptive dynamics when transmission is density-dependent and there is differential migration of infectious poultry

First, we find the endemic equilibrium of the resident. To do this, we set all model equations that describe the infection dynamics to 0. Then we put E^* in terms of I^* using the $\frac{dI}{dt} = 0$ equation (where * denotes the endemic equilibrium):

$$\sigma E^* = (m+\gamma+\delta+\mu)I^*$$
$$E^* = \frac{(m_I+\gamma+\delta+\mu)I^*}{\sigma}$$

We then put H^* in terms of I^* using the $\frac{dH}{dt} = 0$ equation:

$$\lambda I^* - \kappa\psi H^* = 0$$
$$H^* = \frac{\lambda I^*}{\kappa\psi}$$

Next, we use the $\frac{dE}{dt} = 0$ equation to find an expression for S^*:

$$0 = \beta S^* I^* + \epsilon\beta S^* H^* - (\sigma+\mu+m)E^*$$
$$= \beta S^* I^* + \epsilon\beta S^*(\frac{\lambda}{\kappa\psi})I^* - (\sigma+\mu+m)(\frac{m_I+\gamma+\delta+\mu}{\sigma})I^*$$
$$I^* S^*(\beta+\epsilon\beta\frac{\lambda}{\kappa\psi}) = (\sigma+\mu+m)(\frac{m_I+\gamma+\delta+\mu}{\sigma})I^*$$
$$S^*(\beta+\epsilon\beta\frac{\lambda}{\kappa\psi}) = (\sigma+\mu+m)(\frac{m_I+\gamma+\delta+\mu}{\sigma})$$
$$S^* = (\sigma+\mu+m)(\frac{m_I+\gamma+\delta+\mu}{\sigma})\frac{1}{(\beta+\epsilon\beta\frac{\lambda}{\kappa\psi})}$$

Next, we find $\frac{1}{S^*}$:

$$\begin{aligned}
S^* &= (\sigma+\mu+m)(\frac{m_I+\gamma+\delta+\mu}{\sigma})\frac{1}{(}\beta+\epsilon\beta\frac{\lambda}{\kappa\psi})\\
\frac{1}{S^*} &= \frac{\sigma(\beta+\epsilon\beta\frac{\lambda}{\kappa psi})}{(\sigma+\mu+m)(m_I+\gamma+\delta+\mu)}\\
&= \frac{\sigma}{\sigma+\mu+m}[(\beta+\epsilon\beta\frac{\lambda}{\kappa\psi})\frac{1}{m_I+\gamma+\delta+\mu}]\\
&= (\frac{\sigma}{\sigma+\mu+m})(\frac{1}{m_I+\gamma+\delta+\mu})\beta[1+\frac{\epsilon\lambda}{\kappa\psi}]\\
&= \frac{\mathcal{R}_0}{S^{[0]}}
\end{aligned}$$

Thus,

$$S^* = \frac{S^{[0]}}{\mathcal{R}_0} \tag{12}$$

$$S_r^* = \frac{S^{[0]}}{\mathcal{R}_0(\alpha_r)} \tag{13}$$

The differential equations of the mutant strategy that tries to invade the resident population when initially rare are:

$$\begin{aligned}
\frac{dE}{dt} &= \beta S_r^* + \epsilon\beta S_r^* H - \sigma E - \mu E - mE\\
\frac{dI}{dt} &= \sigma E - m_I I - \gamma I - \delta I - \mu I\\
\frac{dH}{dt} &= \lambda I - \kappa\psi H
\end{aligned}$$

In order to find an expression for $\mathcal{R}_i(\alpha_r, \alpha_m)$, which is written as $s_r(m)$ in "The Hitchhiker's Guide to Adaptive Dynamics," or the invasion fitness of the mutant when it tries to invade the resident, we use a next-generation approach.[4] First, we find that the F and V matrices for the next-generation matrix G are:

$$F = \begin{pmatrix} 0 & \beta S_r^* & \epsilon\beta S_r^* \\ 0 & 0 & 0 \\ 0 & 0 & 0 \end{pmatrix}; V = \begin{pmatrix} \sigma+\mu+m & 0 & 0 \\ -\sigma & m_I+\gamma+\delta+\mu & 0 \\ 0 & -\lambda & \kappa\psi \end{pmatrix}$$

Immediately, we recognize that the only difference between the F and V matrices here and the F and V matrices when deriving $\mathcal{R}_0$ above is the replacement of $S^{[0]}$ with S_r^*. Thus, because the rest of the derivation is the same as the above, to arrive at an expression for $\mathcal{R}_i(\alpha_r, \alpha_m)$ we can simply replace $S^{[0]}$ with S_r^*. Thus, because the rest of the derivation is the same as the above, to arrive at an expression for $\mathcal{R}_i(\alpha_r, \alpha_m)$ we can simply replace $S^{[0]}$ with S_r^* in the expression of $\mathcal{R}_0$:

$$\mathcal{R}_i(\alpha_r, \alpha_m) = S_r^*(\frac{\sigma}{\sigma+\mu+m})(\frac{1}{m_I+\gamma+\delta+\mu})\beta[1+\frac{\epsilon\lambda}{\kappa\psi}]$$

$$= S_r^* \frac{\mathcal{R}_0(\alpha_m)}{S^{[0]}}$$

Next, we plug S_r^* into our expression for $\mathcal{R}_i(\alpha_r, \alpha_m)$:

$$\begin{aligned}
\mathcal{R}_i(\alpha_r, \alpha_m) &= S_r^* \frac{\mathcal{R}_0(\alpha_m)}{S^{[0]}} \\
&= \frac{S^{[0]}}{\mathcal{R}_0(\alpha_r)} \frac{\mathcal{R}_0(\alpha_m)}{S^{[0]}} \\
&= \frac{\mathcal{R}_0(\alpha_m)}{\mathcal{R}_0(\alpha_r)} \qquad (14)
\end{aligned}$$

Next, we look for singular strategies (critical points of $\mathcal{R}_i(\alpha_r, \alpha_m)$). We do this by taking the derivative of $\mathcal{R}_i(\alpha_r, \alpha_m)$ with respect to the mutant strategy, then evaluating it at $\alpha_m = \alpha_r$ and setting it to 0:[4]

$$\begin{aligned}
\frac{\partial}{\partial \alpha_m} \mathcal{R}_i(\alpha_r, \alpha_m) &= \frac{\mathcal{R}_0'(\alpha_m)}{\mathcal{R}_0(\alpha_r)} \\
0 &= \frac{\mathcal{R}_0'(\alpha_m)}{\mathcal{R}_0(\alpha_r)} \\
0 &= \frac{\mathcal{R}_0'(\alpha_m = \alpha_r)}{\mathcal{R}_0(\alpha_r)} \\
0 &= \frac{\mathcal{R}_0'(\alpha_r)}{\mathcal{R}_0(\alpha_r)}
\end{aligned}$$

Thus, singular strategies are those virulence strategies, α^*, such that $\mathcal{R}_0' = 0$ since the denominator is positive.

Next, we check the condition for convergence stability of the singular strategy, which is:

$$\begin{aligned}
\frac{\partial}{\partial \alpha_r} \mathcal{R}_i'(\alpha_r, \alpha_m = \alpha_r)\Big|_{\alpha_r = \alpha^*} &< 0 \qquad (15) \\
\frac{\partial}{\partial \alpha_r} \frac{\mathcal{R}_0'(\alpha_r)}{\mathcal{R}_0(\alpha_r)}\Big|_{\alpha_r = \alpha^*} &< 0 \\
\frac{\mathcal{R}_0''(\alpha_r)}{\mathcal{R}_0(\alpha_r)} - \frac{(\mathcal{R}_0'(\alpha_r))^2}{(\mathcal{R}_0(\alpha_r))^2}\Big|_{\alpha_r = \alpha^*} &< 0 \\
\frac{\mathcal{R}_0''(\alpha^*)}{\mathcal{R}_0(\alpha^*)} - \frac{(\mathcal{R}_0'(\alpha^*))^2}{(\mathcal{R}_0(\alpha^*))^2} &< 0 \\
\frac{\mathcal{R}_0''(\alpha^*)}{\mathcal{R}_0(\alpha^*)} &< 0
\end{aligned}$$

In the fourth line, the second term on the L.H.S. equals 0 since $\mathcal{R}_0'(\alpha^*) = 0$ since α^* is a singular strategy (critical point). Thus, for convergence stability, the condition is that $\mathcal{R}''(\alpha^*) < 0$ since the denominator is positive. This is exactly the ESS criterion of the singular strategy, since:[4]

$$\frac{\partial^2 \mathcal{R}_i(\alpha_r, \alpha_m)}{\partial \alpha_m^2}\Big|_{\alpha_r = \alpha_m = \alpha^*} < 0 \qquad (16)$$

$$\frac{\mathcal{R}_0''(\alpha^*)}{\mathcal{R}_0(\alpha^*)} < 0$$

And thus, the ESS criterion is that $\mathcal{R}_0''(\alpha^*) < 0$ since the denominator is positive.

We have shown that the ESS criterion and condition for convergence stability of a singular strategy is the same. Furthermore, α^* such that $\mathcal{R}_0''(\alpha^*) < 0$ are local maxima of $\mathcal{R}_0$ and thus if a virulence strategy is evolutionary stable and convergent stable then it is a local maxima of $\mathcal{R}_0$. In the other direction, if a virulence strategy is a local maxima of $\mathcal{R}_0$ this implies that it is evolutionarily stable and convergent stable. Thus, there is an if-and-only-if condition of local maxima of $\mathcal{R}_0$ and evolutionary stability and convergence stability. Thus, by finding the virulence strategy that maximizes $\mathcal{R}_0$, this is equivalent to finding the singular strategy that is both evolutionarily stable and convergent stable.

Appendix 5: Derivation of endemic equilibrium in markets when transmission is frequency dependent for model of the main results

When transmission is density dependent the unique endemic equilibrium is previously proven in Shuai and van den Driessche.[11]

To prove a unique endemic equilibrium of the the resident when transmission is frequency-dependent, we first put E^* in terms of I^* using the $\frac{dI}{dt} = 0$ equation:

$$\sigma E^* = (m + \gamma + \delta + \mu)I^*$$
$$E^* = \frac{(m + \gamma + \delta + \mu)I^*}{\sigma}$$

We then put H^* in terms of I^* using the $\frac{dH}{dt} = 0$ equation:

$$\lambda I^* - \kappa\psi H^* = 0$$
$$H^* = \frac{\lambda I^*}{\kappa\psi}$$

We then put R^* in terms of I^* using the $\frac{dR}{dt} = 0$ equation:

$$\gamma I^* - (m + \mu)R^* = 0$$
$$R^* = \frac{\gamma I^*}{m + \mu}$$

From the remaining model equations, we then have two equations with two unknowns, S^* and I^*:

$$0 = b - \frac{\beta}{N^*}S^*I^* - \epsilon\frac{\beta}{N^*}S^*H^* - (\mu + m)S^* + m_f S_f$$
$$0 = \frac{\beta}{N^*}S^*I^* + \epsilon\frac{\beta}{N^*}S^*H^* - (\sigma + \mu + m)E^*$$

First, we put N^* in terms of S^* and I^*:

$$\begin{aligned} N^* &= S^* + E^* + I^* + R^* \\ &= S^* + \frac{(m + \gamma + \delta + \mu)I^*}{\sigma} + I^* + \frac{\gamma I^*}{m + \mu} \\ &= S^* + I^*(\frac{m + \gamma + \delta + \mu}{\sigma} + 1 + \frac{\gamma}{m + \mu}) \end{aligned}$$

Next, we use the $\frac{dE}{dt} = 0$ equation to put S^* in terms of I^*:

$$\begin{aligned}
0 &= \frac{\beta}{N^*}S^*I^* + \epsilon\frac{\beta}{N^*}S^*H^* - (\sigma+\mu+m)E^* \\
&= \frac{\beta}{N^*}S^*I^* + \epsilon\frac{\beta}{N^*}S^*\frac{\lambda}{\kappa\psi}I^* - (\sigma+\mu+m)\frac{(m+\gamma+\delta+\mu)}{\sigma}I^* \\
&= \frac{\beta}{N^*}S^* + \epsilon\frac{\beta}{N^*}S^*\frac{\lambda}{\kappa\psi} - (\sigma+\mu+m)\frac{(m+\gamma+\delta+\mu)}{\sigma} \\
&= \frac{\beta}{N^*}S^*(1+\epsilon\frac{\lambda}{\kappa\psi}) - (\sigma+\mu+m)\frac{(m+\gamma+\delta+\mu)}{\sigma} \\
\frac{\beta}{N^*}S^*(1+\epsilon\frac{\lambda}{\kappa\psi}) &= (\sigma+\mu+m)\frac{(m+\gamma+\delta+\mu)}{\sigma} \\
\beta S^*(1+\epsilon\frac{\lambda}{\kappa\psi}) &= (\sigma+\mu+m)\frac{(m+\gamma+\delta+\mu)}{\sigma}N^* \\
\beta S^*(1+\epsilon\frac{\lambda}{\kappa\psi}) &= (\sigma+\mu+m)\frac{(m+\gamma+\delta+\mu)}{\sigma}(S^* + I^*(\frac{(m+\gamma+\delta+\mu)}{\sigma} + 1 + \frac{\gamma}{m+\mu})) \\
\beta S^*(1+\epsilon\frac{\lambda}{\kappa\psi}) &= (\sigma+\mu+m)\frac{(m+\gamma+\delta+\mu)}{\sigma}S^* + (\sigma+\mu+m)\frac{(m+\gamma+\delta+\mu)}{\sigma}I^*(\frac{(m+\gamma+\delta+\mu)}{\sigma} + 1 + \frac{\gamma}{m+\mu}) \\
\beta S^*(1+\epsilon\frac{\lambda}{\kappa\psi}) - (\sigma+\mu+m)\frac{(m+\gamma+\delta+\mu)}{\sigma}S^* &= (\sigma+\mu+m)\frac{(m+\gamma+\delta+\mu)}{\sigma}I^*(\frac{(m+\gamma+\delta+\mu)}{\sigma} + 1 + \frac{\gamma}{m+\mu}) \\
S^*[\beta(1+\epsilon\frac{\lambda}{\kappa\psi}) - (\sigma+\mu+m)\frac{(m+\gamma+\delta+\mu)}{\sigma}] &= (\sigma+\mu+m)\frac{(m+\gamma+\delta+\mu)}{\sigma}I^*(\frac{(m+\gamma+\delta+\mu)}{\sigma} + 1 + \frac{\gamma}{m+\mu}) \\
S^* &= \frac{(\sigma+\mu+m)\frac{(m+\gamma+\delta+\mu)}{\sigma}I^*(\frac{(m+\gamma+\delta+\mu)}{\sigma} + 1 + \frac{\gamma}{m+\mu})}{[\beta(1+\epsilon\frac{\lambda}{\kappa\psi}) - (\sigma+\mu+m)\frac{(m+\gamma+\delta+\mu)}{\sigma}]} \\
S^* &= \Big[\frac{(\sigma+\mu+m)\frac{(m+\gamma+\delta+\mu)}{\sigma}(\frac{(m+\gamma+\delta+\mu)}{\sigma} + 1 + \frac{\gamma}{m+\mu})}{[\beta(1+\epsilon\frac{\lambda}{\kappa\psi}) - (\sigma+\mu+m)\frac{(m+\gamma+\delta+\mu)}{\sigma}]}\Big]I^* \\
S^* &= T^*I^*
\end{aligned}$$

Next we substitute S^* in terms of I^* to put N^* in terms of solely I^*:

$$\begin{aligned}
N^* &= S^* + I^*(\frac{(m+\gamma+\delta+\mu)}{\sigma} + 1 + \frac{\gamma}{m+\mu}) \\
&= TI^* + I^*(\frac{(m+\gamma+\delta+\mu)}{\sigma} + 1 + \frac{\gamma}{m+\mu}) \\
&= I^*\big[T + (\frac{(m+\gamma+\delta+\mu)}{\sigma} + 1 + \frac{\gamma}{m+\mu})\big] \\
&= I^*U
\end{aligned}$$

Finally we put everything into the $\frac{dS}{dt} = 0$ equation and solve for I^*:

$$\begin{aligned}
0 &= b - \frac{\beta}{N^*}S^*I^* - \epsilon\frac{\beta}{N^*}S^*H^* - (\mu+m)S^* + m_fS_f \\
&= b - \frac{\beta}{I^*U}S^*I^* - \epsilon\frac{\beta}{I^*U}S^*\frac{(m+\gamma+\delta+\mu)}{\kappa\psi}I^* - (\mu+m)S^* + m_fS_f \\
&= b - \frac{\beta}{U}S^* - \epsilon\frac{\beta}{U}S^*\frac{(m+\gamma+\delta+\mu)}{\kappa\psi} - (\mu+m)S^* + m_fS_f \\
&= b - S^*[\frac{\beta}{U} + \epsilon\frac{\beta}{U}\frac{(m+\gamma+\delta+\mu)}{\kappa\psi} + (\mu+m)] + m_fS_f \\
&= b - S^*V + m_fS_f \\
&= b - TI^*V + m_fS_f \\
TI^*V &= b + m_fS_f \\
I^* &= \frac{b + m_fS_f}{TV} \qquad (17)
\end{aligned}$$

In markets $b = 0$ so I^* further reduces to:

$$I^* = \frac{m_f S_f}{TV} \tag{18}$$

In order to show that I^* is postive when $\mathcal{R}_0 > 1$ we note that m_f and S_f are positive so the numerator is positive. Now we need to show that the denominator, TV, is positive when $\mathcal{R}_0 \geq 1$. First, we look at T:

$$T = \Big[\frac{(\sigma+\mu+m)\frac{(m+\gamma+\delta+\mu)}{\sigma}(\frac{(m+\gamma+\delta+\mu)}{\sigma} + 1 + \frac{\gamma}{m+\mu})}{[\beta(1+\epsilon\frac{\lambda}{\kappa\psi}) - (\sigma+\mu+m)\frac{(m+\gamma+\delta+\mu)}{\sigma}]}\Big] \tag{19}$$

Because all parameters of the numerator are positive, the numerator is positive. We then look at the denominator:

$$\begin{aligned}
&\beta(1+\epsilon\frac{\lambda}{\kappa\psi}) - (\sigma+\mu+m)(\frac{(m+\gamma+\delta+\mu)}{\sigma}) \\
&= (\frac{1}{\sigma+\mu+m})(\frac{\sigma}{m+\gamma+\delta+\mu})[\beta + \frac{\epsilon\beta\lambda}{\kappa\psi}] - 1 \\
&= (\frac{\sigma}{\sigma+\mu+m})\frac{\beta}{m+\gamma+\delta+\mu} + \frac{\epsilon\beta\lambda}{\kappa\psi(m+\gamma+\delta+\mu)} - 1 \\
&= \mathcal{R}_0 - 1
\end{aligned} \tag{20}$$

Thus, we see that when $\mathcal{R}_0 > 1$ the denominator will be positive. Thus, T is positive when $\mathcal{R}_0 > 1$.

Next we look at V:

$$\begin{aligned}
V &= \frac{\beta}{U} + \epsilon\frac{\beta}{U}\frac{m+\gamma+\delta+\mu}{\kappa\psi} + (\mu+m) \\
&= \frac{\beta}{[T + (\frac{(m+\gamma+\delta+\mu)}{\sigma} + 1 + \frac{\gamma}{m+\mu})]}(1+\epsilon\frac{m+\gamma+\delta+\mu}{\kappa\psi}) + (\mu+m)
\end{aligned} \tag{21}$$

Because T is positive when $\mathcal{R}_0 > 1$ and all parameters are positive, V is also positive when $\mathcal{R}_0 > 1$. Thus, we have shown that $I^* = \frac{m_f S_f}{TV}$ is positive when $\mathcal{R}_0 > 1$, and thus that there is a unique endemic equilibrium when $\mathcal{R}_0 > 1$ when transmission is frequency-dependent.

Appendix 6: Evolutionary stable strategy results for a variation of the main model that considers distinct cohort periods, with cleaning solely between cohorts

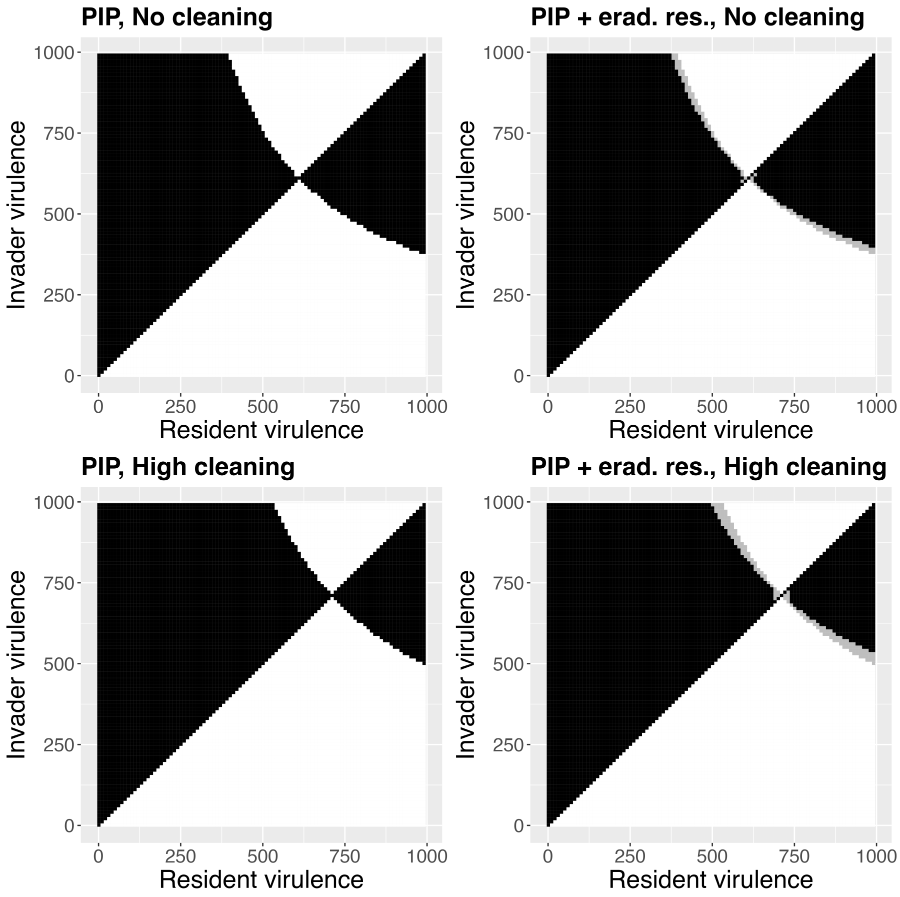

Figure S1: **Pairwise invasibility plots with discrete cohorts** showing success (black, > 2 infected chickens at the end of the simulation) and failure (white, < 1 infected chickens) of invasion by the strategy on the y axis (left column), extended to illustrate resident dynamics (right column, gray areas indicate resident virus persistence alongside invaders with > 2 infected chicken at the end of the simulation) in a context of no cleaning (top row, $\kappa = 0$) and high cleaning (bottom row, a proportion $\kappa = 0.95$ of viral particles cleaned between cohorts). Cleaning selects for an increase in the evolutionary stable virulence strategy. Each cohort generation duration, $T = 90$ days. The transmission-mortality tradeoff parameters used were $c_1 = 1/2300$, $c_2 = 0.45$, $\phi = 10$. Other parameters used were $\sigma = 1/5$ days, $\gamma = 1/5$ days, $\mu = 1/365$ days, $\psi = 1/5$ days, $m = 1/5.5$ days, $m_f = 0.1/120$ days, $S_f = 1$ million. For each cell, the resident strategy was run for 1000 cohort generations, a single infected poultry with the invading virulence strategy was introduced and the simulation was run further for 1000, 10000, or 200000 cohort generations, depending on whether there was clear growth or eradication of the invading strategy.

Industrialized poultry populations may have distinct cohorts, where each cohort of poultry is replaced after some number of days and replaced with a new cohort of completely susceptible poultry.[12] Environmental particles may survive between cohorts, and some proportion of them are removed from cleaning. We create a variation of the model and compare the evolutionary stable strategies using pairwise invasibility plots with and without cleaning. The model equations are:

$$\frac{dS}{dt} = \beta SI - \epsilon\beta SH - \mu S + m_f S_f - mS \tag{22a}$$

$$\frac{dE}{dt} = \beta SI - \epsilon\beta SH - \sigma E - \mu E - mE \tag{22b}$$

$$\frac{dI}{dt} = \sigma E - mI - \gamma I - \delta I - \mu I \tag{22c}$$

$$\frac{dR}{dt} = \gamma I - mR - \mu R \tag{22d}$$

$$\frac{dH}{dt} = \lambda I - \psi H \tag{22e}$$

$$S(nT^+) = 9999 \tag{22f}$$

$$E(nT^+) = 0 \tag{22g}$$

$$I(nT^+) = 0 \tag{22h}$$

$$R(nT^+) = 0 \tag{22i}$$

$$H(nT^+) = \kappa H(nT^-) \tag{22j}$$

Where the first five equations model the dynamics within cohorts and the last five equations model the instantaneous replacement of each cohort with 9999 susceptible poultry and a surviving proportion of environmental particles, $\kappa H(nT^-)$. nT^+ is the time immediately after the nth replacement of the cohort, and nT^- is the time immediately before the nth replacement of the cohort. All model parameters are the same as described in the main text with the exception of κ, which is now the proportion of viral particles cleaned between cohorts.

Our results show that with increased cleaning, the ESS increases for the variation of our model that assumes distinct cohort periods with cleaning between cohorts (Figure S1). This result recapitulates that cleaning will select for increased virulence for industrialized poultry populations.[12]

In the main version of our model that does not assume these distinct cohort periods, we find that the ESS decreases with increased cleaning. Though preliminary, these supplementary results suggest that whether cleaning will select for increased or decreased virulence may depend on whether distinct cohort periods, with cleaning between cohorts, is assumed. Prior theoretical work has also shown that we may expect the mortality rate of propagules (which is affected by cleaning) to become a significant virulence factor in periodically fluctuating environments.[13] However, as described in the discussion of the main text, this assumption of distinct cohort periods may not be appropriate for rural live-poultry markets, which have overlapping, partial replacement of cohorts from many different sellers. Without the assumption of cohorts, cleaning selects for lower virulence, shown in our main results.

References

[1] Jane M Heffernan, Robert J Smith, and Lindi M Wahl. Perspectives on the basic reproductive ratio. *Journal of the Royal Society Interface*, 2(4):281–293, 2005.

[2] Odo Diekmann, Johan Andre Peter Heesterbeek, and Johan AJ Metz. On the definition and the computation of the basic reproduction ratio r 0 in models for infectious diseases in heterogeneous populations. *Journal of mathematical biology*, 28:365–382, 1990.

[3] Pauline Van den Driessche and James Watmough. Reproduction numbers and sub-threshold endemic equilibria for compartmental models of disease transmission. *Mathematical biosciences*, 180(1-2):29–48, 2002.

[4] Åke Brännström, Jacob Johansson, and Niels Von Festenberg. The hitchhiker's guide to adaptive dynamics. *Games*, 4(3):304–328, 2013.

[5] Furaha Chuma, Gasper G Mwanga, and Damian Kajunguri. Modeling the role of wild birds and environment in the dynamics of newcastle disease in village chicken. 2018.

[6] Elifuraha Barnabas Mngumi, Fulgence Ntangere Mpenda, and Joram Buza. Epidemiology of newcastle disease in poultry in africa: systematic review and meta-analysis. *Tropical Animal Health and Production*, 54(4):214, 2022.

[7] Margaret McCarron, Peninah Munyua, Po-Yung Cheng, Thomas Manga, Cathryn Wanjohi, Ann Moen, Anthony Mounts, and Mark A Katz. Understanding the poultry trade network in kenya: Implications for regional disease prevention and control. *Preventive veterinary medicine*, 120(3-4):321–327, 2015.

[8] Henry M Kariithi, Helena L Ferreira, Catharine N Welch, Leonard O Ateya, Auleria A Apopo, Richard Zoller, Jeremy D Volkening, Dawn Williams-Coplin, Darren J Parris, Tim L Olivier, et al. Surveillance and genetic characterization of virulent newcastle disease virus subgenotype v. 3 in indigenous chickens from backyard poultry farms and live bird markets in kenya. *Viruses*, 13(1):103, 2021.

[9] Sophie Molia, Ismaël Ardho Boly, Raphaël Duboz, Boubacar Coulibaly, Javier Guitian, Vladimir Grosbois, Guillaume Fournié, and Dirk Udo Pfeiffer. Live bird markets characterization and trading network analysis in mali: Implications for the surveillance and control of avian influenza and newcastle disease. *Acta tropica*, 155:77–88, 2016.

[10] Tobias O Okeno, Alexander K Kahi, and Kurt J Peters. Characterization of indigenous chicken production systems in kenya. *Tropical animal health and production*, 44:601–608, 2012.

[11] Zhisheng Shuai and P Van den Driessche. Global dynamics of cholera models with differential infectivity. *Mathematical biosciences*, 234(2):118–126, 2011.

[12] Carly Rozins and Troy Day. The industrialization of farming may be driving virulence evolution. *Evolutionary Applications*, 10(2):189–198, 2017.

[13] Sébastien Lion and Sylvain Gandon. Evolution of class-structured populations in periodic environments. *Evolution*, 76(8):1674–1688, 2022.
