# Supplementary material for: Markets as drivers of selection for highly virulent poultry pathogens
Source: Nat Commun. 2024 Jan 19;15:605. doi: 10.1038/s41467-024-44777-3 (PMC10799013; doi:10.1038/s41467-024-44777-3)
Supplement: Supplementary file 3 — Reporting Summary [file 41467_2024_44777_MOESM3_ESM.pdf]

nature portfolio

Corresponding author(s): Justin Sheen

Last updated by author(s): Dec 15, 2023

Reporting Summary

Nature Portfolio wishes to improve the reproducibility of the work that we publish. This form provides structure for consistency and transparency in reporting. For further information on Nature Portfolio policies, see our Editorial Policies and the Editorial Policy Checklist.

Statistics

For all statistical analyses, confirm that the following items are present in the figure legend, table legend, main text, or Methods section.

n/a	Confirmed	
☐	☒	The exact sample size (*n*) for each experimental group/condition, given as a discrete number and unit of measurement
☐	☒	A statement on whether measurements were taken from distinct samples or whether the same sample was measured repeatedly
☒	☐	The statistical test(s) used AND whether they are one- or two-sided<br>*Only common tests should be described solely by name; describe more complex techniques in the Methods section.*
☒	☐	A description of all covariates tested
☒	☐	A description of any assumptions or corrections, such as tests of normality and adjustment for multiple comparisons
☒	☐	A full description of the statistical parameters including central tendency (e.g. means) or other basic estimates (e.g. regression coefficient) AND variation (e.g. standard deviation) or associated estimates of uncertainty (e.g. confidence intervals)
☒	☐	For null hypothesis testing, the test statistic (e.g. *F*, *t*, *r*) with confidence intervals, effect sizes, degrees of freedom and *P* value noted<br>*Give P values as exact values whenever suitable.*
☒	☐	For Bayesian analysis, information on the choice of priors and Markov chain Monte Carlo settings
☒	☐	For hierarchical and complex designs, identification of the appropriate level for tests and full reporting of outcomes
☒	☐	Estimates of effect sizes (e.g. Cohen's *d*, Pearson's *r*), indicating how they were calculated

Our web collection on statistics for biologists contains articles on many of the points above.

Software and code

Policy information about availability of computer code

Data collection	We conducted all simulations using the R programming language (Version 4.2.3) within the RStudio IDE (Version 2023.03.0+386). For testing of the epidemiological dynamics we used the "deSolve" package (Version 1.35). Simulated data, code, and results that support the findings of this study are openly available in GitHub at http://www.github.com/jsheen/marketVirEvol. All code has also been deposited in the Zenodo database under accession code https://doi.org/10.5281/zenodo.10373146.
Data analysis	We conducted all simulations using the R programming language (Version 4.2.3) within the RStudio IDE (Version 2023.03.0+386). Simulated data, code, and results that support the findings of this study are openly available in GitHub at http://www.github.com/jsheen/marketVirEvol. All code has also been deposited in the Zenodo database under accession code https://doi.org/10.5281/zenodo.10373146.

For manuscripts utilizing custom algorithms or software that are central to the research but not yet described in published literature, software must be made available to editors and reviewers. We strongly encourage code deposition in a community repository (e.g. GitHub). See the Nature Portfolio guidelines for submitting code & software for further information.

Data

Policy information about availability of data

All manuscripts must include a data availability statement. This statement should provide the following information, where applicable:

- Accession codes, unique identifiers, or web links for publicly available datasets
- A description of any restrictions on data availability
- For clinical datasets or third party data, please ensure that the statement adheres to our policy

The data on the average duration that poultry stay in live-poultry markets in this study have been deposited in the Zenodo database under accession code https://doi.org/10.5281/zenodo.10403195. Informed consent was obtained and approved by the Princeton University IRB office (IRB Record Number 12138).

Research involving human participants, their data, or biological material

Policy information about studies with human participants or human data. See also policy information about sex, gender (identity/presentation), and sexual orientation and race, ethnicity and racism.

Reporting on sex and gender	N/A
Reporting on race, ethnicity, or other socially relevant groupings	N/A
Population characteristics	In this study, we do not report any characteristics collected of the poultry traders, solely the number of days until chickens of each trader were sold.
Recruitment	Live poultry traders who participated in the survey were recruited on a voluntary basis. As such, there may be self-selection of participants, though it is unclear how this would bias our estimate of the umber of days until chickens are sold by traders.
Ethics oversight	IRB office of Princeton University (IRB record number 12138)

Note that full information on the approval of the study protocol must also be provided in the manuscript.

Field-specific reporting

Please select the one below that is the best fit for your research. If you are not sure, read the appropriate sections before making your selection.

☒ Life sciences ☐ Behavioural & social sciences ☐ Ecological, evolutionary & environmental sciences

For a reference copy of the document with all sections, see nature.com/documents/nr-reporting-summary-flat.pdf

Life sciences study design

All studies must disclose on these points even when the disclosure is negative.

Sample size	Live poultry traders were surveyed on a voluntary basis (convenience sample) resulting in a sample size of 130 traders. This is a large sample size, and is sufficient since the surveyed traders were representative of the heterogeneity found in two areas of high trade in Madagascar (Antananarivo and Toamasina).
Data exclusions	No data concerning the rate of poultry trade was excluded.
Replication	N/A as no experiments were conducted in the study.
Randomization	Randomization was not applicable since no RCT experiment was done to collect the data (the average number of days poultry stayed in markets before being sold).
Blinding	Blinding of researchers or traders during data collection was not performed due to efficiency of surveying. It was also unclear whether there was a clear reason for a bias of under or over-reporting the variable of interest to emerge without blinding. The published data are anonymized.

Reporting for specific materials, systems and methods

We require information from authors about some types of materials, experimental systems and methods used in many studies. Here, indicate whether each material, system or method listed is relevant to your study. If you are not sure if a list item applies to your research, read the appropriate section before selecting a response.

Materials & experimental systems

n/a	Involved in the study
☒	☐ Antibodies
☒	☐ Eukaryotic cell lines
☒	☐ Palaeontology and archaeology
☒	☐ Animals and other organisms
☒	☐ Clinical data
☒	☐ Dual use research of concern
☒	☐ Plants

Methods

n/a	Involved in the study
☒	☐ ChIP-seq
☒	☐ Flow cytometry
☒	☐ MRI-based neuroimaging

Plants

Seed stocks	N/A
Novel plant genotypes	N/A
Authentication	N/A
